# Treatment of burns in the first 24 hours: simple and practical guide by answering 10 questions in a step-by-step form

**DOI:** 10.1186/1749-7922-7-13

**Published:** 2012-05-14

**Authors:** Ziyad Alharbi, Andrzej Piatkowski, Rolf Dembinski, Sven Reckort, Gerrit Grieb, Jens Kauczok, Norbert Pallua

**Affiliations:** 1 Department of Plastic and Hand Surgery, Burn Centre Medical Faculty, RWTH Aachen University Hospital, Pauwelsstr 30, Aachen, 52074, Germany; 2 Department of Plastic and Reconstructive Surgery, azM University Hospital, Maastricht, Netherland; 3 Department of Operative Intensive Care, Medical Faculty, RWTH Aachen University, Aachen, Germany; 4 Department of Anaesthesia and Intensive Medicine, St. Elisabeth Hospital Geilenkirchen, Geilenkirchen, Germany

**Keywords:** Burn care, Burn surgery, Burn unit, Burn resuscitation, Burn care guidelines

## Abstract

Residents in training, medical students and other staff in surgical sector, emergency room (ER) and intensive care unit (ICU) or Burn Unit face a multitude of questions regarding burn care**.** Treatment of burns is not always straightforward. Furthermore, National and International guidelines differ from one region to another. On one hand, it is important to understand pathophysiology, classification of burns, surgical treatment, and the latest updates in burn science. On the other hand, the clinical situation for treating these cases needs clear guidelines to cover every single aspect during the treatment procedure. Thus, 10 questions have been organised and discussed in a step-by-step form in order to achieve the excellence of education and the optimal treatment of burn injuries in the first 24 hours. These 10 questions will clearly discuss referral criteria to the burn unit, primary and secondary survey, estimation of the total burned surface area (%TBSA) and the degree of burns as well as resuscitation process, routine interventions, laboratory tests, indications of Bronchoscopy and special considerations for Inhalation trauma, immediate consultations and referrals, emergency surgery and admission orders. Understanding and answering the 10 questions will not only cover the management process of Burns during the first 24 hours but also seems to be an interactive clear guide for education purpose.

## Introduction

During a rotation to the emergency room (ER), surgical sector or burn unit, residents under training should pay attention to the pathophysiology and classification of burns, treatment, and the latest updates in burn science including burn injury prognosis [1]. Managing burn cases in the first 24 hours represents one of the biggest challenges in burn care and will indeed reflect the degree of morbidity and mortality. Therefore, a guide for treatment during the first 24 hours can be very helpful. A lot of trusted guidelines exist regarding this point such as the American Burn Association guidelines for referral criteria to burn centres and also operation guidelines in the burn unit. Furthermore, it should be noted that the International society for Burn injuries (ISBI) served a good purpose regarding the education and set several guidelines with the World Health Organisations and many European organisations including the European Burn Association, German Society for Burn Treatment and British Burn Association for the treatment of Burn injuries.

This practical guide is drawn to make it easy for any trainee, medical students and staff to understand the basic principles of management that should be carried out in each burn case during the first 24 hours. Any trainee should understand indeed his/her responsibility for these unique patients and should identify the management process in comprehensive way. This does not only mean covering of all wounds but also to bring the patient to his or her normal status including the psychological, social and of course the physical aspect.

## Objective

This article has been primarily written for education purposes. We believe that good and clear information will indeed enhance the quality of treatment even without big facilities. The target group is any physician, surgeon, trainee in training, interns, medical students and personnel who are responsible for burn patients in surgical sector, emergency room (ER) and intensive care unit (ICU) or Burn Unit.

## Methods

A clear guide has been structured for the above target group, which includes 10 questions that should be asked and well answered to cover the treatment of burn patients in the first 24 hours. Herein, the following questions should be taken in consideration:

1. Does the patient meet the criteria for injuries requiring referral to the Burn Unit?

2. How to perform the Primary Survey and Secondary Survey?

3. How to estimate the total burned surface area (%TBSA) and the degree of burns?

4. What are the main aspects of Resuscitation?

5. What are the routine interventions that should be performed for each case of burn injury during admission to the Burn Unit?

6. What kind of laboratory tests should be done?

7. Does the patient have Inhalation Injury and is Bronchoscopy indicated for all patients?

8. What kind of consultations should be carried out immediately?

9. Does the patient need Emergency Surgery or not?

10. What kind of admission orders should be written?

Furthermore, this paper does not only state a guideline to be followed but also explains every point and takes in consideration that many hospitals around the world do not have a specialised burn unit and, thus most of the treatment process occurs in the emergency room (ER). Furthermore, international guidelines regarding burn treatment have been also reviewed in the literature.

10 questions as practical guide:

1. ***Does the patient meet the criteria for injuries requiring referral to the Burn Unit?***

A clear answer should be given in the pre-hospital setting. This must be well performed by the referral person or the transporting physician. It is not meant that a patient with burn injury should immediately be moved to a burn unit. In the case of a burn centre not being able to accept a patient, the initial treatment process can also be conducted in the emergency room (ER) until the transport to the burn unit takes place.

The main criteria for referral to a burn unit include the following [2]:

Second and third degree burns greater than 10% TBSA in patients younger than 10 years and older than 50 years.

Second and third degree burns greater than 20%.

Third degree burns greater than 5%.

Burns to face, hands, feet, genitalia, perineum and major joints.

Electrical burns (including lightning injury)

Chemical burns

Inhalation injury

Patients with pre-existing conditions

Circumferential third degree burns to extremity or chest

Burns involving concomitant trauma with a great risk of morbidity and mortality (i.e. explosion trauma).

2. ***How to perform the Primary Survey and Secondary Survey?***

The burn injury itself has a secondary role in the moment of ***primary survey***. Directly on admission Advanced Trauma Life Support (ATLS) guidelines must be performed and the following points must be checked:

**A**irway: Early recognition of airway compromise followed by prompt intubation can be live saving [3]. If there is soot in the mouth consider early intubation even if the patient is breathing normally.

**B**reathing: Determine if the patient is moving air or not.

**C**irculation: Obtain appropriate vascular access and a monitor device to control heart rate and blood pressure.

**D**isability: Detect if there are any other manifestations including fractures and deformities, abdominal injury or neurological deficit.

**E**xposure: The patient should be completely exposed and should be out of clothes. Exposure of all orifices must be conducted in this part.

**F**luid resuscitation: A mainstay in the treatment. This point is discussed in the third question after the calculation of the total burned surface area (%TBSA) but the guidelines of Acute Trauma Life Support (ATLS) should be followed in order to maintain the circulation process.

Note that a child is prone to hypothermia due to its high surface to volume ratio and low fat mass. Ambient temperature should be from 28° to 32°C (82° to 90°F). The patient’s core temperature must be kept at least above 34°C.

***Secondary survey*** is designed as a burn-specific survey. It is performed during admission to the burn unit. Full history should be approached including:

Examination of the cornea is important as well as the ear in case of explosion trauma. A systemic overview should be performed in this phase including a fast run on the *abdomen*, *genital region*, *lower and upper limbs* (think: X-Ray C-Spine, Thorax, and Pelvic). If the patient is a child, look for signs of abuse.

Detection of the mechanism of injury.

Time of injury.

Consideration of abuse [4].

Height and weight.

Possibility of carbon monoxide intoxication based on the history of burns in a closed area as well as the presence of soot in mouth and nose [5].

Facial burns.

3. ***How to estimate the total burned surface area (%TBSA) and the degree of burns?***

**Figure 1:**
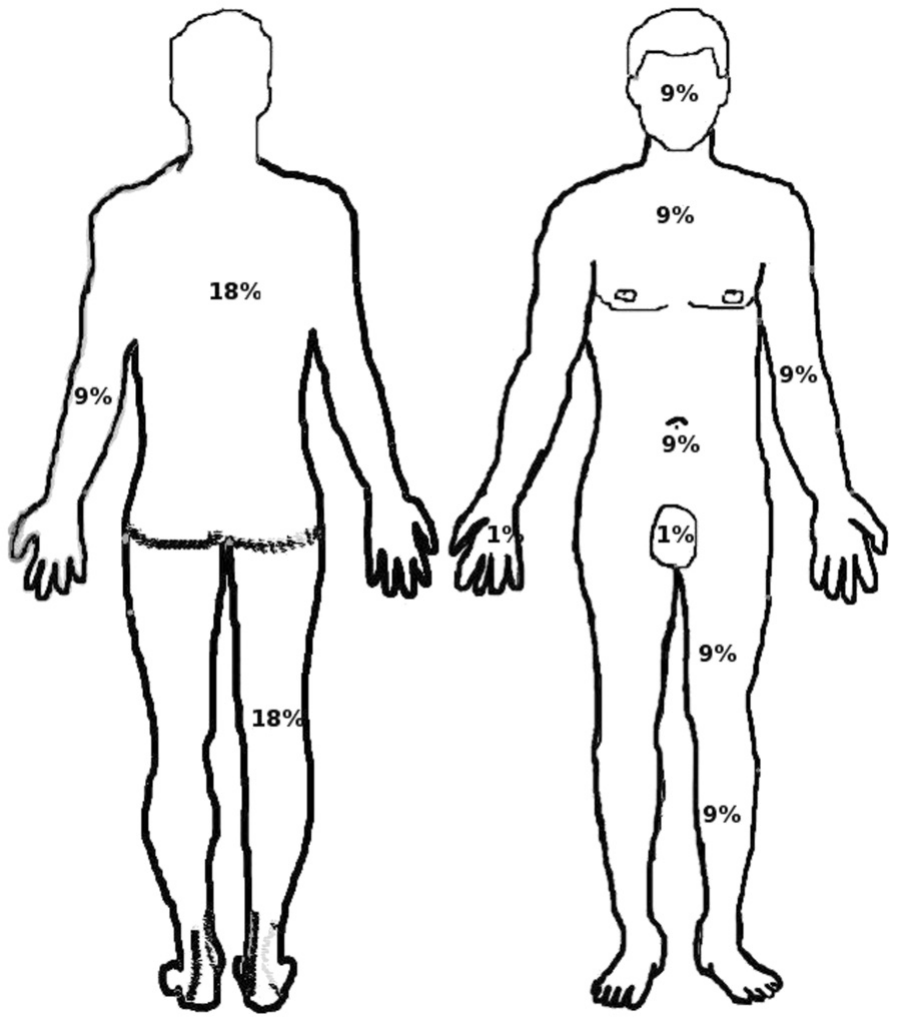
Rule of nines: This figure shows the different parts of the body that equal 9% of the body surface area (i.e. complete upper thigh = 9%, complete lower thigh = 9%, complete leg = 18%).

Total body surface area (TBSA) is an assessment measure of skin burns. As shown in Figure 1, in adults the "***rule of nines***" is used to determine the total percentage of the burned area for each major section of the body [6,7].However, this rule cannot be used in pediatric burns. The ***Lund-Browder chart*** is one of the most accurate methods to estimate not only the size of the burn area but also the burn degree in each part. The use of this chart has shown an easy access and fast readability in the clinical practice as well as its use in pediatric burns [7]. It is available in many centres and also available online. Note that an internet address has been added at the end of this article to make it accessible for education purposes. Accurate estimation must be performed in order to estimate the amount of intravenous fluids, referral indications to the burn unit and indication of surgery as well as the estimation of prognosis.

The degree of burns is calculated to estimate the prognosis as well as the type of treatment and consequently the type of surgery that should be conducted. Burns are classified to:

First degree burns: typical redness and pain of the affected skin. Minor epithelial damage occurs without formation of blisters. Typically occurs with sunburns.

Superficial second degree burns: complete epithelial damage and only papillary dermal damage occurs. This degree leaves no neurovascular damage. Thus, it causes pain, bleeds and presents with blisters. Epithelial repair occurs within 14 days. It mostly leaves no scars after healing. Sometimes discoloration stays.

Deep second degree burns: complete epithelial damage and damage of the reticular dermis present. It results in neurovascular damage. Thus, it generally presents without bleeding or sensation and appears white in colour. Blisters can also be present but are bigger than in superficial second degree burns. Healing can occur but takes longer than 14 days and results in scars.

**Figure 2:**
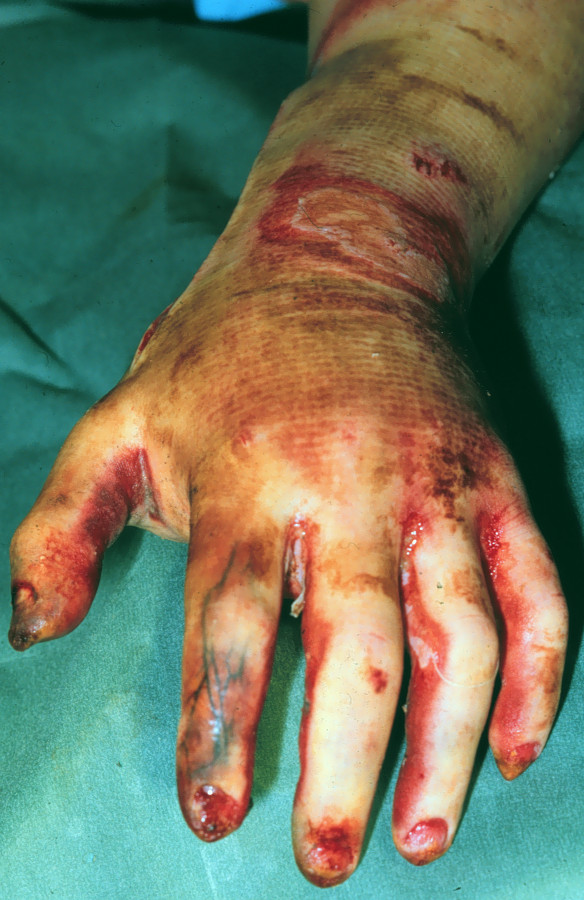
**Third degree burns (Note the thrombotic vessels formation)**.

Third degree burns: involving the epidermis, dermis and subcutaneous tissue. The skin appears leathery consisting of thrombotic vessels (Figure 2).

Forth degree burns (*debatable*): it is a third degree burn with involvement of the underlying fascia, muscles and even bones.

Superficial burn injury (First degree).

Superficial partial-thickness burns (Superficial second degree).

Deep partial-thickness burns (Deep second degree).

Full-thickness burns (Third degree).

Fourth degree burns (debatable classification as some references do not support this degree [1]).

4. ***What are the main aspects of Resuscitation?***

Calculation of the total burned surface (% TBSA) area is essential in this part. Charles Baxter, MD, at Parkland Hospital, Southwestern University Medical Centre, designed in the 1960s [8,9] the ***Parkland formula*** to calculate the fluid needs for the first 24 hours. Although many modifications of this formula have been proposed this formula is still one of the easiest ways to calculate the fluid volume for burn patients.

4mL×Patient's body weight×TBSA=Volume to be given in the first 24 hours

*50% of this volume is infused in the first 8 hours, starting from the time of injury, and the other 50% is infused during the last 16 hours of the first day*.

The type of fluid administration is a debatable question. Lactated Ringer has been commonly used and is even used up to date. On the other hand, many centres suggest balanced electrolyte solutions like Ringer-acetate to prevent the high dose administration of lactate. According to our experience and to the best of our knowledge, we believe that balanced electrolyte solutions are a safe option and therefore they are recommended in our centre. Furthermore, specific burn populations usually require higher resuscitation volumes sometimes as much as 30-40% higher (close to 5.7 mL/kg/%TBSA) than predicted by the Parkland formula [10,11]. Klein et al have suggested that patients today are receiving more fluid than in the past. Their purpose was to find significant predictors of negative outcomes after resuscitation. They concluded that higher volumes equalled a higher risk for complications, i.e. lung-complications [12,13]. These results support that fluid overload in the critical hours of early burn management may lead to unnecessary oedema [14].

Overall, the use of Parkland formula is just a process of estimation. Clinically, fluid needs of an individual, after the use of any suggested formula, should be at least monitored by several important factors such urine output, blood pressure and central venous pressure. An important point and considered to be the goal in fluid resuscitation is to maintain a urine output of approximately 0.5 ml/kg/h in adults and between 0.5 and 1.0 ml/kg/h in patients weighing less than 30 kg [15]. Failure to meet these goals should be addressed with gentle upward corrections in the rate of fluid administration by approximately 25% [16].

Due to the capillary leak, most burn centres advise not to use colloids and other blood products within the first 24 hours [17]. If used in the early phase (up to 12 h), it can lead to a prolonged tissue oedema and consecutive lung complications. Furthermore colloids are not associated with an improvement in survival, and are therefore more expensive than crystalloids [18]. Liberati et al advocated that there is no evidence that blood products (including human albumin) reduce mortality when compared with cheaper alternatives such as saline [19].

***Maintenance dose*** is provided after the first 24 hours. It can be calculated as follows [1,20]:

$\begin{array}{l} 100\;\text{ml}/\text{kg}:\;\text{for the first}\;10\;\text{kg}\\ 50\;\text{ml}/\text{kg}:\;\text{for the second}\;10\;\text{kg}\\ 20\;\text{ml}/\text{kg}:\;\text{every kilogram above}\;20\;\text{kg} \end{array}$

Special considerations for children:

***Modified Parkland Formula*** is used for this category of patients as follows [1,21]:

$\begin{array}{c} 4\;\text{mL}\times \text{Patient's body weight}\times \text{TBSA}\times \text{Maintenance fluid}\\ =\\ \text{Volume to be given in the first 24 hours} \end{array}$

5. ***What kind of routine interventions should be performed for each case of burns during admission to the Burn Unit?***

Injured patients differ in term of burns size and depth. Pre-existing conditions play an important role in this phase. ***Central venous catheter*** and ***arterial line*** are indicated if the patient is hemodynamically unstable or if frequent blood gas analysis is required. Furthermore, ***nasogastric tube*** and ***urinary catheter*** are indicated in patients with 20% TBSA or more. Nasogastric tube will initiate immediate feeding and decrease the possibility of ileus or aspiration. Urinary catheter that is equipped with a temperature probe is preferred.

Before washing the patients, ***swabs for microbiological examination*** should be taken from different areas including burn areas, mouth, nose and the inguinal area. It should be made clear that ***the patient is washed*** properly with warm water and then re-evaluated regarding the total burned surface area (TBSA) as well as the degree of burns. A definite evaluation of the total burned surface area (TBSA) can only be made when the patient is washed completely and the wounds can be judged properly. In this phase, indication for surgery is made including escharotomy, debridement and in certain situations skin grafting. This point will be discussed in the 9^th^ question.

6. ***What kind of laboratory tests should be done?***

Basic laboratory tests include the following:

Complete blood count (CBC) and Arterial blood gas (ABG) analysis,

Urea and Electrolytes (U&E),

Prothrombin time (PT) / Partial thrombin time (PTT) and International Normalized Ration (INR),

Sputum Culture and Sensitivity,

Creatine Kinase (CK) and C-reactive protine (CRP),

Blood glucose,

Urine drug test,

Human chorionic gonadotropin (B-HCG): if the patient is female,

Albumin test.

Thyroid values and myoglobin measures.

7. ***Does the patient have Inhalation Injury and is Bronchoscopy indicated for all patients?***

Burns occurring in closed areas and all burns that are affecting the head are subjected to inhalation injury [22,23]. If Carbon monoxide (CO) intoxication is suspected, perform arterial blood gas (ABG) analysis to detect carboxyhemoglobin (COHb), immediate supply of 100% oxygen, chest X-Ray and discuss the possibility of hyperbaric oxygen (HBO) therapy. COHb higher than 20% or cases presented with neurological deficits are absolute indications for HBO, whereas COHb amounts of 10% and higher are seen as relative indications for HBO [24]. Overall, intubated burn patients provide a good access for bronchoscopy. In this case, fiberoptic bronchoscopy can be used to evaluate the extent of airway oedema and the inflammatory process that is caused by any form of inhalation injury including the carbon monoxide (CO) intoxication [22,23]. On the other hand, the role of bronchoscopy is debatable in terms of the therapeutic aspect as well as its invasive procedure.

8. ***What kind of consultations should be carried out immediately?***

Depending on the secondary survey, several consultations may be necessary. In case of facial burns, consult:

Otolaryngology (**ENT**) department: to exclude burns of the upper airway, laryngeal oedema or in case of explosion rupture of the tympanic membrane.

Ophthalmology: to exclude erosion or ulceration of the cornea.

Follow the same procedure as performed in the primary survey. As guided by the Advance Trauma Life Support (ATLS), consult or re-consult if already performed:

Trauma surgery,

Abdominal surgery and

Neurosurgery.

9. ***Does the patient need Emergency Surgery or not?***

*Debridement:*

The term ''Debridement'' is not merely a surgical procedure. Debridement can be performed by surgical, chemical, mechanical, or autolytic procedures. Surgical modalities including early tangential excision (***necrectomy***) of the burned tissue and early wound closure primarily by skin grafts has led to significant improvement in mortality rates and substantially lower costs in these patients [25,26]. Furthermore, in some circumstances, ***escharotomy*** or even ***fasciotomy*** should be performed.

Indications of surgical debridement:

Dermal substitutes or matrices can be used if a large burn area exists. Here are some examples:

Note that in many occasions, an immediate coverage of wounds cannot be achieved. In this case, a temporary coverage is favoured. After stabilization of patient and wound bed, a planned reconstruction takes place to close wounds permanently. In this point, some methods can be performed including:

1. Deep second degree burns.

2. Burns of any type, that are heavily contaminated

3. Third degree circumferential burns with suspected compartment syndrome (think of: *Escharotomy*)

4. Circumferential burns around the wrist (think of: *Carpal tunnel release*)

Benefits of surgical debridement:

1. To reduce the amount of necrotic tissue (beneficial for prognosis)

2. To get a sample for diagnostic purposes (if needed).

Complications of debridement:

1. Pain.

2. Bleeding.

3. Infection.

4. Risk of removal of healthy tissue.

Contraindications:

1. Low body core temperature below 34°C.

2. Cardiovascular and respiratory system instability.

Any trainee should be aware of the following terms:

Tangential excision: Tangential excision of the superficial (burned) parts of the skin

Epifascial excision: This technique is reserved for burns extending at least to the subcuticular level.

Subfascial excision: indicated when burns extend vey deep and reach the fascia and muscles. It is needed only in special cases.

**Figure 3:**
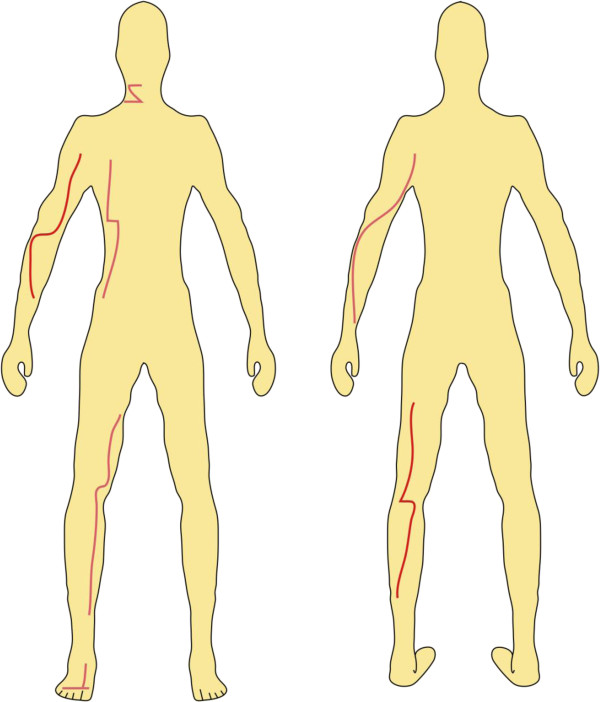
**Escharotomy lines: Example of typical ways to incise the eschar. **Note that the incisions should be made horizontally when crossing a joint.

Escharotomy: Indicated for third-degree and second degree deep dermal circumferential burns. This is used to prevent a soft tissue compartment syndrome, due to swelling after deep burn. An escharotomy is performed by making an incision through the eschar to expose the fatty tissue below. This can be illustrated in Figure 3. Note that escharotomy lines on the thumb and little finger, as an international standard, should be always performed on the radial side and not on the ulnar side. Escharotomy incisions for the index finger, middle finger and ring finger are performed along the ulnar side.

Fasciotomy: Fasciotomy is a limb-saving procedure when used to treat acute compartment syndrome. An incision is made in the skin that extends into the fascia where it will relieve pressure. Note that Carpal Tunnel Syndrome (CTS) can result from the circumferential burns around the wrist by consecutive swelling.

**Figure 4:**
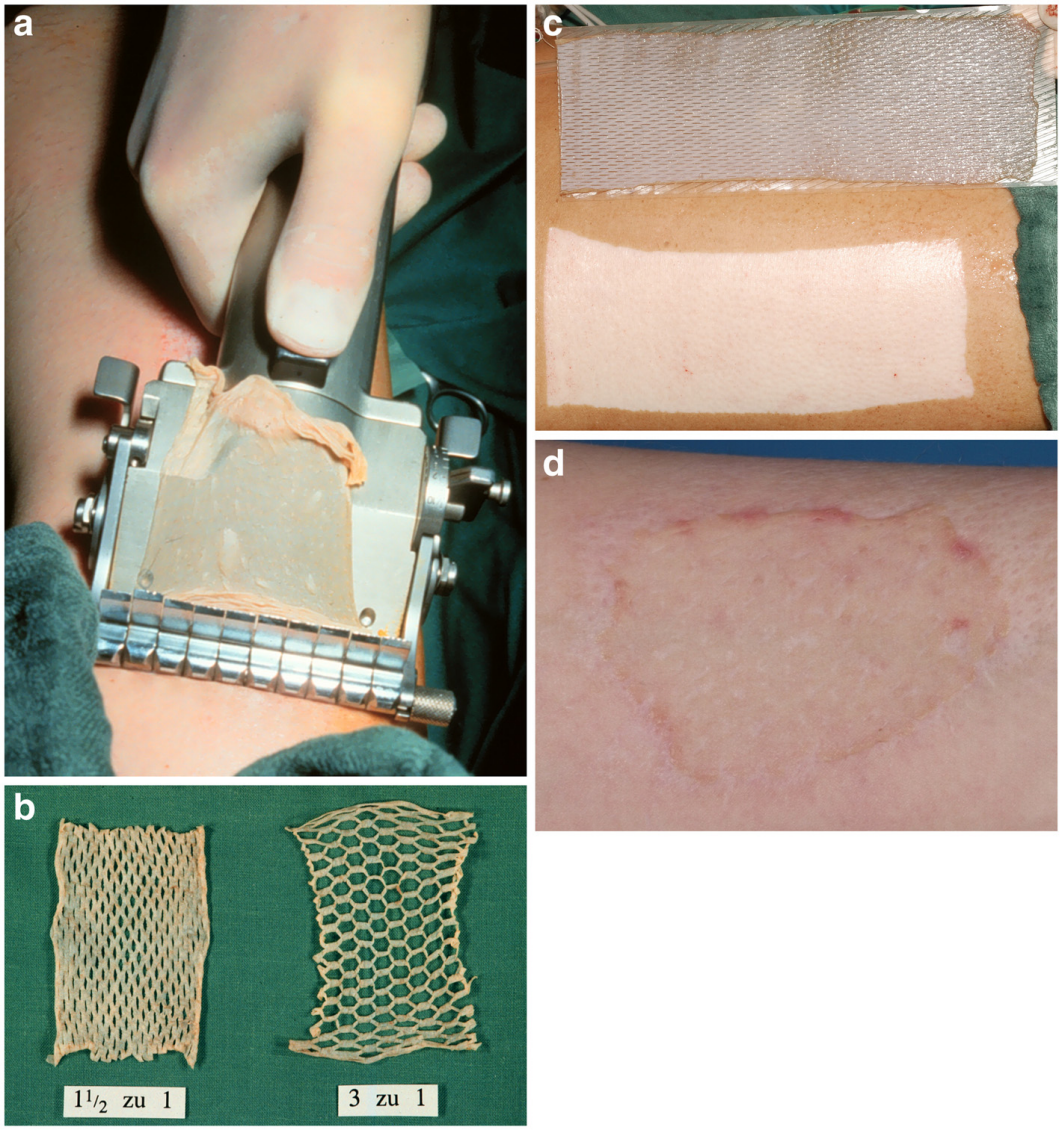
**a: Harvesting a skin graft with a dermatome, b: MESH skin graft with different sizes, c: the donor site after harvesting the skin graft, d: the appearance of the skin graft after its attachment to the Recipient area (3 Weeks later)**.

**Figure 5:**
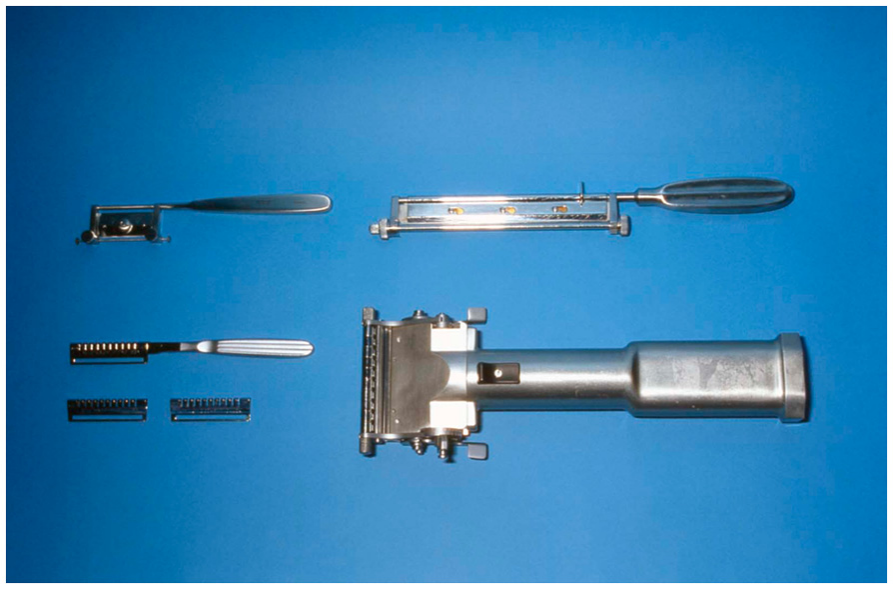
**This figure shows the most widely used instruments for skin debridement and harvesting of the graft**.

After any selected procedure from the above category, the resulted wound should be covered. Autografts, i.e. ***split thickness skin grafts*** (autologous skin transfer), remain the mainstay of treatment for many patients (Figure 4a-d and 5).

Biobrane: Biosynthetic wound dressing constructed of a silicone film with a nylon fabric.

Suprathel: Innovative skin substitute made of polylactide for the treatment of superficial dermal wounds especially the superficial second degree burns.

Alloderm: Cultured and processed dermis used under skin graft to reproduce the layered structure of dermis and epidermis in a graft

Integra: Bilayer wound matrix comprised of porous matrix of cross-linked bovine tendon collagen and glycosaminoglycan and a semi-permeable polysiloxane (silicone) layer. Must be used in a two-step-procedure [27].

Matriderm: Three dimensional matrix consisting of collagen and elastin. Its use guides autologous cells for the construction of a "neo-dermis" [28,29]. Can be used in a single-step as well as in a two-step-procedure.

Allografts: Cadaver Skin used for temporary cover.

Xenografts: Graft taken from other species (bovine of swine) can be used as temporary cover.

10. ***What kind of admission orders should be written?***

Routine admission orders include:

Vital signs: Continuous monitoring of Heart rate, Blood pressure, Pulse pressure, Respiratory rate, Temperature and Central venous pressure.

Documentation of allergies

Diet: Nil per os (NPO) if burn more than 30% during the first 24 hours. Nasogastric tube will initiate immediate feeding and decrease the possibility of ileus or aspiration.

I.V. fluids: follow the Parkland formula.

Decubitus precautions.

Consultation: Psychiatry or Psychology (only if patient is awake).

Multivitamins and Traces: Vitamine C, ZnSo4, Selenium and Vitamine E.

Tetanus prophylaxis.

Ulcer prophylaxis.

Analgesia: the choice is dependent on burn size, depth, age and other trauma factor such as blunt trauma and fractures.

Additional medications (for mechanically ventilated adults with smoke inhalation injury): nebulized heparin sulfate mixed in 3 ml normal saline every 4 hours and 3 ml 20% nebulized N-acetylcysteine plus 0.5 ml albuterol sulfate every 4 hours for 7 days [30].

## Discussion

Several guidelines regarding burn management exist. This includes those guidelines setup by organisations and by clinicians or researchers in the field. Kis et al searched the literature between 1990 and 2008 and retrieved 546 citations, of which 24 were clinical practice guidelines on the general and intensive care of burn patients. All major burn topics were covered by at least one guideline, but no single guideline addressed all areas important in terms of outcomes [31]. For example, Alsbjoern B et al structured a guideline for treatment but that was mainly concentrating on wound treatment rather than the comprehensive way [32].

One of the most renown and used guidelines have been set up by the International Society for Burn Injuries (ISBI) and the American Burns Association. The IBSI works together with the World Health Organisation and, thus enhances the education process concerning burn injury treatment in the developing world. The American Burn Association guidelines are considered one of the most reliable guidelines and are even followed and trusted by other big associations and societies like the South African Burn Society or the Australian and New Zealand Burn Association.

The criteria for transfer to a burn centre may differ between the above stated organisations. However, the criteria setup by the American Burn association represents the most widespread so far and are also fully supported by the American College of Surgeons [33-36]. In Europe, a workgroup of burn centres in German speaking countries (DAV) developed very well established guidelines for the treatment as well as the referral to a burn unit, which are accepted by the German Society for Burn Treatment (DGV), as well as the Austrian and the Swiss Burn Societies [37]. On the other hand, these guidelines don’t discuss all aspects of treatment in the acute phase. There is no doubt that these guidelines and other factors including the development of advanced technologies in burn care enhanced the quality of treatment for burn patients in the last decades. However, many of these guidelines are made primarily for plastic surgeons and represent too much information regarding wound management and long term planning of surgical reconstruction.

In contrast to the above stated guidelines this paper discusses the first 24 hours in Burns and includes not only the surgical treatment but also a polytrauma protocol as well as a basic intensive care treatment plan for those patients.

This paper is written without intention to cover the therapy of electrical and chemical burns. We believe that electrical and chemical burns need a special evaluation and treatment that differs from thermal burns. Overall, thermal burns are common if compared to the last 2 types and, thus this guide concentrates on thermal burns. Furthermore, this paper takes in consideration that the information must be simple but also effective with good explanation just to be easily reached in a time frame as short as possible.

## Conclusion

Understanding and answering the above stated 10 questions will not only cover the management process of Burns during the first 24 hours but also should be an interactive clear guide for education purpose. Burn cases can extremely differ and, thus trainee, medical students and personnel in surgical sector, emergency room (ER) and intensive care unit (ICU) or Burn Unit face a multitude of questions regarding these critically ill patients. We found that this method serves good purposes and increases not merely the quality of treatment but also enhances education. Therfore it was good reason and positive motivation for us to structure another 10 questions as a clear guide that cover the treatment of burns after the first 24 hours until discharge.

### Recommendations

**Advanced Burn Life Support (ABLS) Course** by American Burn Association provides guidelines in the assessment and management of the burn patient during the first 24 hours post injury. To date, this course is of great importance like the **Advanced Trauma Life Support (ATLS)** course, which is provided by the American College of Surgeons and many centres around the world. We should declare that there is no financial or commercial relationship between authors and those organisations providing these types of courses.

### Recommendation of further sources for education purpose

Abbreviated burn severity index (ABSI) / Belgian outcome in burn injury (BOBI)

*Lund and Browder chart for calculating the percentage of total body surface area burnt:*http://www.tg.org.au/etg_demo/etg-lund-and-browder.pdf

*internet-based burns chart:*http://www.burnschart.com

Harris Benedict Equation / Curreri Formula for calorie needs.

## Competing interests

Authors declare that they have no competing interests.

## Authors' contributions

ZA carried out the design of the review, participated in literature review and prepared the manuscript. AP participated in the preparation of illustrations and figures of the review, preparation of the manuscript and literature review. SR, GG and JK participated in preparation of the draft and manuscript review. RD and NP contributed to critical discussion of the draft, preparation of the draft and manuscript review. All authors read and approved the final manuscript.
